# Association of *PDGFRB*, *VEGFR2*, and *TGFB1* Gene Polymorphisms with Nintedanib Efficacy and Adverse Effects in Patients with Progressive Pulmonary Fibrosis

**DOI:** 10.3390/ijms27177546

**Published:** 2026-08-23

**Authors:** Natasa Djurdjevic, Marija Dusanović Pjevic, Mihailo Stjepanovic, Nikola Colic, Sanja Dimic-Janjic, Jelena Jankovic, Nikola Maric, Andrej Zecevic, Milica Terzic, Aleksa Golubovic, Ivan Milivojevic, Maja Omcikus, Branislav Ilic, Katarina Lukic, Snjezana Mijatovic, Milka Grk

**Affiliations:** 1 Clinic for Pulmonology, University Clinical Center of Serbia, 11000 Belgrade, Serbia; natalidjurdjevic@yahoo.com (N.D.); mihailostjepanovic@gmail.com (M.S.);; 2 Faculty of Medicine, University of Belgrade, 11000 Belgrade, Serbia; 3 Institute of Human Genetics, Faculty of Medicine, University of Belgrade, 11000 Belgrade, Serbia; marija.dusanovic-pjevic@med.bg.ac.rs; 4 Centre for Radiology, University Clinical Center of Serbia, 11000 Belgrade, Serbia; 5 Clinic for Allergology and Immunology, University Clinical Center of Serbia, 11000 Belgrade, Serbia

**Keywords:** progressive pulmonary fibrosis, nintedanib, pharmacogenetics, polymorphisms, PDGFRB, VEGFR2, TGFB1, adverse effects

## Abstract

Progressive pulmonary fibrosis (PPF) comprises interstitial lung diseases characterized by progressive fibrosis and lung function decline. Nintedanib slows disease progression by inhibiting FGFR, VEGFR, and PDGFR signaling, but treatment response varies among patients. We investigated whether *VEGFR2*, *PDGFRB*, and *TGFB1* gene variants are associated with nintedanib treatment outcomes, including changes in pulmonary function, diffusing capacity and adverse effects. This prospective study included 75 patients with PPF diagnosed according to ATS/ERS/JRS/ALAT criteria and treated with nintedanib at the Clinic of Pulmonology, University Clinical Center of Serbia. Spirometry and diffusing capacity were assessed at baseline and after 6 and 12 months. Genotyping of *VEGFR2* rs2071559 and rs1870377; *PDGFRB* rs2302273, rs2229562, and rs246395; and *TGFB1* rs1800470 was performed using TaqMan^®^ assays. Carriers of the *PDGFRB* rs2302273 A allele more frequently experienced gastrointestinal adverse events (45.2% vs. 20.5%; *p* = 0.022; OR 3.2, 95% CI 1.156–8.866) and diarrhea (38.7% vs. 15.9%; *p* = 0.026; OR 3.3, 95% CI 1.129–9.869). Patients with the *PDGFRB* rs246395 TT genotype more frequently had a ≥15% decline in DLCO after one year (46% vs. 22%; *p* = 0.038; OR 3.0, 95% CI 1.045–8.394). No significant associations were observed for the analyzed *VEGFR2* or *TGFB1* variants. These findings suggest that *PDGFRB* variants may be associated with nintedanib treatment outcomes, although confirmation in larger cohorts is needed.

## 1. Introduction

Pulmonary fibrosis is a pathological process that can occur due to various underlying diseases, among which a notable place is occupied by systemic autoimmune diseases associated with interstitial lung disease (SARD-ILD). The diagnosis and treatment of these patients represent a complex challenge in modern pulmonology due to the notable heterogeneity in the pathophysiology and clinical course [1,2]. Interstitial pulmonary disease (ILD) is a frequent extra-articular manifestation of rheumatological autoimmune diseases, with an overall prevalence of around 40%. The highest frequency is observed in systemic sclerosis (47%) and in idiopathic inflammatory myopathies (41%), while a lower prevalence is noticed in Sjögren’s syndrome (17%), rheumatoid arthritis (11%), and systemic lupus erythematosus (6%) [1]. Despite the initial injury cause, which is different for various autoimmune diseases, the progressive pulmonary fibrosis (PPF) phenotype derived in these patients is quite homogeneous and is characterized by quick decrease in Forced Vital Capacity (FVC) and poor outcome [3,4]. Approximately 57.3% of patients develop a progressive phenotype, compared to an estimated 18–32% in patients with interstitial lung diseases other than idiopathic pulmonary fibrosis (IPF) patients [5,6,7]. After the initial injury of epithelial cells and a change in the composition of the epithelium in the form of the proximalization of the distal lungs, a large amount of cytokines such as TGFβ, PDGF, CTGF and endothelin-1 are produced, which triggers inflammation and activates fibroblast progenitors [2,8,9]. In further maintaining inflammation in ILD associated with autoimmune diseases, autoantibodies from the underlying disease play a key role [3,8,10,11]. Prolonged inflammation driven by cytokines such as TGFβ, as a central mediator in this process, along with IL-6, IL-13, and growth factors such as PDGF, induces the transformation of fibroblasts into myofibroblasts—cells capable of contraction and excessive collagen synthesis [12,13]. As the repair of the injury is inadequate, the creation of collagen exceeds its breakdown, which leads to its accumulation and consequent fibrosis [8]. Neovascularization in the fields of fibrosis is most likely additionally enhanced by the effect of VEGF [14]. In the later stages, the inflammatory signals weaken, while the fibrotic drive becomes autonomous. A positive feedback loop occurs between the cells and the created extracellular matrix, which leads to further progression of fibrosis [15,16,17].

PPF is a rare disease, with an estimated incidence of 6.7 cases per 100,000 individuals in Europe [18]. Treatment is extremely complex and previously included the use of immunosuppressive drugs such as MMF, CYC and AZA with supportive corticosteroid therapy [19]. Today, the drug nintedanib is used in treatment, which has been shown to be a superior therapy in patients with PPF [20]. Nintedanib achieves its antifibrotic effects regardless of the cause of progressive pulmonary fibrosis and the cause of the initial injury [21]. Simple oral administration of 150 mg of the drug per day prevents the fall of FVC. Relatively frequent side effects require dose reduction in 33.1% of patients, while discontinuation of therapy is necessary in 19.6% of subjects according to the INBUILD study [22]. Nintedanib is a small intracellular tyrosine kinase inhibitor that competitively binds to the ATP-binding site of multiple kinases, thereby blocking downstream intracellular signaling cascades. It primarily inhibits receptor tyrosine kinases involved in the pathogenesis of interstitial lung disease (ILD), including PDGFR-α and -β, VEGFR-1, -2 and -3, and FGFR-1, -2 and -3 [23].

The development of new biomarkers, modern imaging diagnostic techniques and the introduction of innovative therapies, such as antifibrotic drugs, provide new hope for improving treatment outcomes and the quality of life of patients with systemic sclerosis-associated interstitial lung disease (SSc-ILD) and other forms of autoimmune pulmonary fibrosis [24]. Previous pharmacogenetic studies have suggested that genetic variability may contribute to interindividual differences in nintedanib treatment outcomes. In patients with IPF, the *DSP* rs2076295 variant has been associated with nintedanib treatment outcomes, while HLA-C*06:02 has been associated with an increased risk of nintedanib-induced gastrointestinal adverse reactions; in contrast, no significant association with treatment response has been reported for *MUC5B* rs35705950 [25,26,27]. However, the available pharmacogenetic evidence remains limited and heterogeneous, with previous studies evaluating different genetic variants and clinical outcomes. Despite the established involvement of VEGFR, PDGFR, and TGFβ signaling in fibrotic processes and the direct inhibition of VEGFR and PDGFR by nintedanib, the contribution of genetic variability within these pathways to treatment outcomes remains insufficiently investigated. To the best of our knowledge, the selected *VEGFR2*, *PDGFRB*, and *TGFB1* polymorphisms have not previously been evaluated in relation to nintedanib treatment outcomes in patients with PPF. Investigating these variants may therefore provide preliminary evidence for potential pharmacogenetic markers of treatment response and tolerability and establish a basis for further studies in larger cohorts.

## 2. Results

The study included 75 participants, 51 (68%) females and 24 (32%) males, with an average age of 61.9±11.4 years. The study cohort predominantly comprised patients with systemic sclerosis-associated ILD (33, 44.0%) and rheumatoid arthritis-associated ILD (17, 22.7%). Less frequent underlying diseases included Sjögren’s syndrome-associated ILD (7, 9.3%), dermatomyositis-associated ILD (5, 6.7%), undifferentiated connective tissue disease-associated ILD (5, 6.7%), systemic lupus erythematosus-associated ILD (3, 4.0%), polymyositis-associated ILD (3, 4.0%), hypersensitivity pneumonitis (1, 1.3%), and sarcoidosis (1, 1.3%).

At baseline, there were no statistically significant differences between patients with and without disease progression after one year of treatment in demographic or functional parameters. Age was comparable between groups (67.5 (59.75–72) vs. 64 (54–71.5) years, *p* = 0.25). Similarly, baseline pulmonary function tests including FEV1, FVC, FEV1/FVC ratio, DLCO, and KCO did not differ significantly between groups. Six-minute walk test and CT-derived alveolar and reticular scores were also similar at baseline (all *p*-values > 0.05), Table 1, suggesting that the groups were clinically well matched prior to follow-up.

Significant differences in longitudinal lung function decline were observed between groups. Progressors demonstrated a greater reduction in FEV1 and FVC at 6 and 12 months compared with non-progressors, as expected. At 6 months, median FEV1 decline was 9.19% (1.35–19.03) in progressors versus 0% (−5.88–4.1) in non-progressors (*p* < 0.001). A similar pattern was observed for FVC decline (11.22 (6.05–21.02) vs. −2 (−11.49–1.57), *p* < 0.001).

At 12 months, FEV1 and FVC decline remained significantly greater in progressors, with a median FVC decline of 16.53% (11.92–23.89) compared with −5% (−16.17–1.73) in non-progressors (*p* < 0.001). Between 6 and 12 months, a smaller but still significant difference in FVC decline was observed between groups (5.01% (0–13.07) vs. 0% (−4.42–4.42), *p* = 0.012).

The proportion of patients experiencing ≥10% decline in lung function was markedly higher among progressors. At 6 months, 57.14% of progressors versus 4.91% of non-progressors experienced ≥10% FVC decline (OR 25.78 (95% CI 5.36–123.99), *p* < 0.001). Similar findings were observed for FEV1 decline thresholds (Table 2). Overall, these findings indicate a consistent and clinically meaningful separation between groups based on longitudinal lung function trajectories, rather than baseline disease severity. DLCO decline did not differ significantly between groups at any time point.

Adverse effects were registered in 29 (38.7%) patients, of whom 23 (30.7%) had gastrointestinal AE, 19 (25.3%) had diarrhea, six (8%) had nausea and six (8%) had hepatotoxicity. Female patients had AE more frequently than male patients (52.9% vs. 8.3%; *p* < 0.001), including gastrointestinal AE (41.2% vs. 8.3%; *p* = 0.004; OR: 7.7; 95% CI 1.632–36.323) and diarrhea (35.3% vs. 4.2%; *p* = 0.004; OR: 12.545; 95% CI 1.563–100.707). The frequency of side effects did not differ between patients with and without disease progression at one-year follow-up, indicating no evident relationship between treatment tolerability and subsequent disease progression in this cohort.

Genotype and allele distributions for the analyzed polymorphisms are represented in Table 3. All analyzed polymorphisms were found to be in Hardy–Weinberg equilibrium (HWE) in the entire study cohort.

Patients who were *PDGFRB* rs2302273 polymorphism A allele carriers more frequently had gastrointestinal AE (45.2% vs. 20.5%; *p* = 0.022; OR: 3.2; 95% CI 1.156–8.866) and diarrhea (38.7% vs. 15.9%; *p* = 0.026; OR: 3.3; 95% CI 1.129–9.869). These results were significant after using a logistic regression analysis adjusted for age and sex for gastrointestinal AE and diarrhea respectively (*p* = 0.042; OR: 3.086; 95% CI 1.043–9.125 and *p* = 0.047; OR: 3.204; 95% CI 1.013–10.137).

Patients with the TT genotype for *PDGFRB* rs246395 polymorphism more frequently exhibited ≥15% DLCO decline after one year of nintedanib therapy (46% vs. 22%; *p* = 0.038; OR: 3; 95% CI 1.045–8.394). This result was significant after using a logistic regression analysis adjusted for age and sex (*p* = 0.025; OR: 3.474; 95% CI 1.167–10.342).

*PDGFRB* (rs2229562, rs246395, rs2302273) and *VEGFR2* (rs1870377, rs2071559) gene haplotype analysis was performed using HaploView software. In patients with PPF, haplotypes TTG, CCG, CCA, TTA, CTA, and CTG within the *PDGFRB* gene were present, the frequencies of which were 59.7%, 15.4%, 9.3%, 7.6%, 5.1% 2.2% respectively. A haplotype block was present between rs2229562 and rs246395 polymorphisms (Supplementary Figure S1). Within *VEGFR2*, we observed TA, TG, AG and AA haplotypes with frequencies of 38.5%, 36.9%, 12.5% and 12.2%, respectively. These two polymorphisms did not form a haplotype block (Supplementary Figure S2). *PDGFRB* and *VEGFR2* haplotypes were not associated with treatment outcome or the occurrence of AE (Supplementary Tables S2 and S3).

## 3. Discussion

The development of nintedanib began more than 25 years ago. It was developed primarily as an antitumor agent and first introduced in the treatment of non-small-cell lung cancer and ovarian carcinoma [28]. Subsequently, its antifibrotic potential was recognized, which led to the use of this drug as an antifibrotic [14,28]. The first studies examining its antifibrotic potential were based on the hypothesis that nintedanib inhibits signaling pathways known to be involved in the pathogenesis of idiopathic pulmonary fibrosis. As promising results were obtained, further research was continued [29,30]. Under in vitro conditions, it was demonstrated that nintedanib indeed exerts an antifibrotic effect on lung fibroblasts through the inhibition of PDGFR, VEGFR, and FGFR [14,31]. Due to the favorable results achieved in IPF therapy, further research has focused on the application of this therapy to progressive pulmonary fibrosis, a significant disease manifestation in patients with systemic sclerosis [32]. Nintedanib was first approved for use in the treatment of IPL in 2014, and subsequently for the treatment of pulmonary manifestations of SSc and, finally, for PPF [33,34].

Several studies have investigated circulating, molecular and pharmacogenomic biomarkers as potential predictors of nintedanib efficacy and toxicity. However, despite promising preliminary findings involving pathways related to VEGF, PDGF, and FGF signaling, no biomarker has yet achieved sufficient validation for routine clinical use [35,36].

Patients with PPF show considerable variability in both therapeutic response and tolerance to nintedanib, with adverse events resulting in dose adjustments or treatment interruption [37,38]. Therefore, the development of predictive genetic biomarkers would be valuable for clinical use [38]. Since single nucleotide polymorphisms remain constant throughout life, genetic testing represents a rapid, reliable, and relatively simple approach for pre-treatment patient stratification. Such biomarkers could help predict therapeutic response, identify patients at risk of toxicity, and support personalized dose adjustment [38,39]. Despite these advantages, only a limited number of studies have investigated the pharmacogenetics of nintedanib [25,26,27,40].

Although the *MUC5B* rs35705950 polymorphism is recognized as a major genetic risk factor for IPF development, two independent studies demonstrated no significant association between this variant and response to nintedanib therapy in IPF patients [26,27]. In the Czech population, the *DSP* rs2076295 polymorphism, previously associated with IPF susceptibility, was found to affect treatment with both nintedanib and pirferidone. According to this study, G allele carriers have lower mortality than IPF patients with the TT genotype who were treated with nintedanib [26]. Mocci et al. reported that carriers of the HLA-C*06:02 allele had a significantly increased risk of nintedanib-induced adverse events, particularly gastrointestinal toxicity, with an approximately 12-fold-higher risk of diarrhea compared to non-carriers. The authors proposed that this association may reflect an HLA-mediated immune mechanism involving excessive IL-23-driven inflammation and intestinal immune dysregulation [25].

This is the first study to examine the association between *PDGFRB*, *VEGFR2*, and *TGFB1* polymorphisms and the efficacy and side effects of nintedanib in patients with PPF.

*PDGFRB* polymorphism rs2302273 is located in the 5′UTR region, while rs2229562 is located in the 3′UTR region, and thus could impact the transcriptional activity of the gene [41,42]. Meanwhile, polymorphism rs246395 within the same gene is located in exone 19, and this c2601T>C is silent. Although silent mutations are historically considered insignificant, it has been proven in later years that these variants could impact the structure and stability of mRNA and translation efficacy, and could disrupt the process of splicing or protein folding [43]. We found that carriers of the *PDGFRB* rs2302273 A allele more frequently exhibited gastrointestinal (GI) AE that included diarrhea, vomiting and nausea. It is yet unknown how nintedanib induces these side effects, although authors over the years suggested a few mechanisms [25,44]. Kato et al. suggested that one of the mechanisms could be the inhibition of the receptors by itself. According to Mocci et al., it is possible that the inhibition of VEGFR could cause intestinal ischemia, while the inhibition of FGFR could disturb intestinal homeostasis, similarly to EGFR inhibitors [25]. It is known that PDGFB and TGFβ promote tenascin C expression during inflammation and promote cell migration, remodeling, and intestinal barrier protection in a mouse model, leading to the assumption that the inhibition of PDGF signaling could potentially impair mucosal repair [45]. This connection could potentially explain why patients with certain allelic variants within the *PDGFRB* itself could be more susceptible to slower regeneration of the intestinal mucosa and, thus, gastrointestinal side effects. Female sex was associated with a higher incidence of adverse events in our cohort, which is in agreement with the findings of Hoffmann-Vold et al. [46]. However, Jovanović et al. reported male sex as an independent predictor of adverse events, suggesting that the relationship between sex and antifibrotic drug tolerability remains uncertain [47]. After adjustment for age and sex in the multivariable logistic regression model, both the rs2302273 polymorphism and female sex remained independent predictors of GI AE. Our results suggest that the *PDGFRB* rs246395 TT genotype may be associated with a less favorable response to nintedanib therapy, reflected by a ≥15% decline in DLCO after one year of treatment. However, to the best of our knowledge, no previous studies have specifically investigated the impact of this polymorphism on treatment outcomes with tyrosine kinase inhibitors in IPF, PPF, or malignant diseases.

VEGFR2, also known as kinase insert domain receptor (KDR), is another target for nintedanib [23]. Polymorphism rs1870377 is a missense variant, located in *VEGFR2* exon 11 [48]. According to Marin et al., the change in the amino acid (p.Gln472His) in the fifth NH2-terminal IgG-like domain of the extracellular region of the receptor leads to a decrease in the affinity of VEGFR for VEGF [49]. Variant rs2071559 is located in the promoter region of the gene [48]. Substitution of c-604A>G disrupts the E2F binding site and causes a decrease in *VEGFR2* expression [49]. People with the CC genotype show a lower concentration of soluble VEGFR in blood, and due to less soluble VEGFR that is able to bind VEGF, this growth factor is free to stimulate angiogenesis via membrane receptors [48]. In our study, rs1870377 and rs2071559 polymorphisms within the *VEGFR2* gene did not affect the outcome of nintedanib treatment or AE occurrence. Although it has been proposed that patients carrying the rs1870377 TT and rs2071559 GG combination have stronger VEGF stimulation, we did not observe a significant association between *VEGFR2* genotype combinations or haplotypes and nintedanib treatment outcomes [48]. We observed that none of the patients with the rs1870377 A allele and rs2071559 AA genotype combination (*n* = 9) had more than a 10% decrease in FVC(L) after one year of treatment, but this result was not statistically significant (*p* = 0.054).

Although no studies have examined the effects of these polymorphisms on the outcome of nintedanib treatment in patients with PPF, their influence on the outcome of malignant disease therapy with other selective and nonselective tyrosine kinase receptor blockers, such as sunitinib, sorafenib, pazopanib, and apatinib, has been evaluated [49,50,51]. Conducted studies showed conflicting results of the impact of rs1870377 and rs2071559 *VEGFR2* polymorphism on sorafenib treatment outcome. While Escudier et al. and Scartozzi et al. reported better survival rates in patients with the rs2071559 GG genotype/G allele, respectively, in the Chinese population, A allele carriers had longer overall survival [52,53,54]. These studies also showed inconsistent results regarding the rs1870377 *VEGFR2* polymorphism, ranging from a protective role for both alleles to conclusions that this variant has no impact on treatment outcomes [52,53,54]. These results could be explained by the different genetic origins of the populations included in these studies. We should also emphasize that while Scartozzi et al. and Zheng et al. included patients with hepatocellular carcinoma, Escudier et al. conducted a study with patients with renal cell carcinoma [52,53,54]. Treatment with sunitinib is significantly influenced by the rs1870377 and rs2071559 polymorphisms, according to George et al.; however, this was not confirmed in other studies [51,55,56]. Additionally, pazopanib therapy in patients with renal cell carcinoma was not affected by these two polymorphisms, although this conclusion should be interpreted with caution due to the extremely small number of patients [51]. An even smaller number of studies dealt with the influence of *PDGFRB* polymorphisms on the response to tyrosine kinase inhibitor therapy. Qin et al. reported no correlation between sorafenib AE and *PDGFRB* polymorphisms rs2229562 and rs2302273. Although Garcia et al. reported that the *PDGFRB* rs246395 polymorphism correlated with increased pathway activation and poorer survival in colorectal cancer, these findings should be interpreted cautiously, as the analysis was performed on tumor-derived rather than germline DNA, which may complicate the interpretation of the true constitutional frequency and biological significance of the detected polymorphisms [57].

The post hoc power analysis indicated a statistical power of 73.83% for detecting a medium effect size (w = 0.30), which is below the conventionally targeted level of 80%. This moderate statistical power, together with the relatively small sample size, may have limited the ability to detect modest genetic effects and increased the risk of false-negative findings, while multiple comparisons may have increased the possibility of false-positive associations. Therefore, given the exploratory and hypothesis-generating nature of this study, the observed associations should be interpreted with caution and require validation in larger, independent cohorts.

Several statistically significant associations were accompanied by relatively wide 95% confidence intervals, particularly those involving female sex, adverse events, and genetic variants. This limited precision likely reflects the relatively small overall sample size, small genotype subgroups, and the limited number of events in some categories. In addition, all analyzed polymorphisms were in Hardy–Weinberg equilibrium in the entire study cohort. Given the absence of a control group, HWE cannot be interpreted in relation to PPF susceptibility, while the low frequency or absence of certain genotypes may reflect allele frequencies and the limited sample size.

The present study has several limitations. The relatively small sample size, limited number of events in some genotype subgroups, and multiple comparisons may have affected the precision and robustness of the observed associations. In addition, the absence of a control group precludes conclusions regarding the role of the analyzed variants in susceptibility to PPF. Future studies should include larger, independent and preferably multicenter cohorts, with replication of the identified associations and, where possible, functional analyses to clarify the biological relevance of the investigated variants.

## 4. Materials and Methods

### 4.1. Study Design and Participants

This study was designed as a prospective study. Patients who were treated at the Clinic for Pulmonology, University Clinical Center of Serbia, between 2025 and 2026 were enrolled. The study included 75 patients diagnosed with PPF according to the ATS/ERS/JRS/ALAT criteria [58]. All enrolled patients had received treatment with nintedanib for more than six months at the Clinic for Pulmonology, University Clinical Center of Serbia. Only adult patients (≥18 years) were included.

All participants were thoroughly informed about the aims of the study and signed written informed consent prior to participation. The study was approved by the Ethics Committee of the University Clinical Center of Serbia (Approval No. 1088/2; 29 May 2025) and by the Head of the Institute of Human Genetics, Faculty of Medicine, University of Belgrade. The exclusion criterion was confirmed malignant lung disease. Demographic data for each patient were recorded at the time of diagnosis.

### 4.2. Pulmonary Function Assessment

Pulmonary function tests were performed for all patients at treatment initiation and during scheduled follow-up visits throughout the course of the disease and therapy, in accordance with the routine monitoring of patients with PPF. Spirometry was performed for all study participants at baseline, after 6 months of treatment, and after 12 months of treatment. The spirometric parameters evaluated included forced vital capacity (FVC), forced expiratory volume in one second (FEV1), and the FEV1/FVC ratio. Spirometric measurements were recorded both as absolute values (mL) and as percentages of the predicted values (% predicted).

Diffusing capacity testing was performed in all patients at baseline, after six months of treatment and after 12 months of treatment, as an indicator of alveolar-capillary membrane function. The diffusion parameters assessed included the diffusing capacity of the lung for carbon monoxide (DLCO) and the transfer coefficient (KCO).

The six-minute walk test (6MWT) was conducted indoors along a straight 30 m corridor. The 6MWT was performed with all patients at baseline, after six months of treatment and after 12 months of treatment. The distance walked within six minutes was recorded while patients walked at a self-selected pace, with rest periods allowed as needed.

### 4.3. High-Resolution Computed Tomography Assessment

High-resolution computed tomography (HRCT) of the chest was used to determine the alveolar and interstitial scores. All patients included in the study had an HRCT scan of the chest before study enrollment. HRCT of the chest was performed using a multidetector CT scanner during full inspiration. Images were reconstructed with thin sections (≤1.5 mm) using a high-spatial-frequency (lung) kernel. All scans were reviewed on dedicated workstations with standard lung window settings. Image analysis was performed independently by experienced thoracic radiologists. The extent of parenchymal abnormalities was assessed using a semi-quantitative scoring system based on lobar involvement. Each lung was divided into five lobes (right upper, middle, and lower lobes; left upper and lower lobes). For each lobe, the extent of parenchymal involvement was visually estimated and scored separately for alveolar abnormalities (ground-glass opacities and consolidation) and fibrotic abnormalities (reticulation, traction bronchiectasis, and honeycombing). The percentage of involvement in each lobe was graded using the following scale: 0: no involvement, 1: <5%, 2: 5–25%, 3: 26–50%, 4: 51–75%, 5: >75%. The scores from all five lobes were summed to obtain a total alveolar score (range: 0–25) and a total fibrotic score (range: 0–25). A higher score indicates more extensive disease involvement.

### 4.4. Assessment of Adverse Effects and Disease Progression

Data on adverse drug reactions were collected and evaluated throughout the treatment period based on patient-reported symptoms, clinical observations during follow-up visits, and biochemical laboratory analyses. The adverse effects monitored included gastrointestinal events (diarrhea, anorexia, vomiting, and nausea), hepatotoxicity (elevated AST and ALT levels), bone marrow toxicity (thrombocytopenia, leukopenia, and pancytopenia), prolonged bleeding, thromboembolic events, nephrotoxicity (serum urea and creatinine levels and the presence of proteinuria), and cardiovascular adverse effects.

Disease progression was defined based on the correlation between pulmonary function test results and HRCT findings. Patients who experienced a decline in FVC (%) of more than 10% during the one-year follow-up were classified as having progressive disease. Disease progression was assigned a value of 1, whereas the absence of disease progression was assigned a value of 0.

### 4.5. Molecular Genetic Analysis

Molecular genetic analyses were carried out at the Institute of Human Genetics, Faculty of Medicine, University of Belgrade. Genomic DNA was isolated from peripheral blood leukocytes using a standard salting-out procedure [59], while its concentration and purity were evaluated spectrophotometrically. Genotyping of polymorphisms in the *VEGFR2* (rs2071559, C__15869271_10 and rs1870377, C__11895315_20), *PDGFRB* (rs2302273, C___2599411_1_; rs2229562, C___3220594_10 and rs246395, C___7507227_10) and *TGFB1* (rs1800470, C__22272997_10) genes was conducted using TaqMan^®^ SNP Genotyping Assays (Applied Biosystems, Foster City, CA, USA) on a 7500 Real-Time PCR System (Applied Biosystems). Real-time PCR allelic discrimination reactions were performed using 4× CAPITAL™ qPCR Probe Mix, low ROX (Biotechrabbit GmbH, Berlin, Germany), according to the manufacturer’s instructions. Each reaction contained 0.5 µL of genomic DNA at a concentration of 10–50 ng/µL, with an A260/A280 absorbance ratio of 1.8–2.0. The same thermal cycling conditions were applied to all TaqMan^®^ SNP Genotyping Assays. The thermal cycling protocol consisted of a pre-PCR read at 60 °C for 1 min, an initial holding stage at 95 °C for 10 min, 40 cycles of denaturation at 95 °C for 15 s and annealing/extension at 60 °C for 1 min, and a final post-PCR read at 60 °C for 1 min. A non-template control (NTC) was included in each PCR run. Allele calling was performed using 7500 Software v2.0.6 (Applied Biosystems). All genotype calls were manually verified by inspection of the amplification curves for each individual sample and the allelic discrimination plot to confirm appropriate clustering of the samples. Detailed genomic characteristics, allele frequencies, and available evidence regarding the functional relevance of the analyzed polymorphisms are provided in Supplementary Table S1 [41,57,60,61].

### 4.6. Statistical Analysis

Continuous variables are presented as mean ± standard deviation (SD) when normally distributed, or as median with range for non-normally distributed data. The distribution of numerical variables was assessed using both graphical methods (histograms, boxplots, and Q–Q plots) and statistical tests, including the Shapiro–Wilk and Kolmogorov–Smirnov tests, together with measures of skewness, kurtosis, and the coefficient of variation. Depending on the data distribution, the Student’s T test, Mann–Whitney test, or Wilcoxon test was performed, where appropriate, for continuous variables. Categorical variables are expressed as absolute frequencies and percentages [*n* (%)]. Differences in categorical variables were evaluated using the Chi-square test or Fisher’s exact test, as appropriate. Variables showing statistical significance in the univariate analysis were subsequently included in a multivariate logistic regression model. The results are presented as odds ratios (ORs) with corresponding 95% confidence intervals (95% CIs) and *p*-values. Haplotype analysis was performed using HaploView 4.2 software. Haplotype blocks were evaluated by using the “Confidence Intervals LD” method [62]. The initial sample size estimation was based on the expected frequency of PPF and indicated a minimum sample of 43 patients for a 95% confidence level and a precision of 0.025. This calculation was not specifically designed to assess statistical power for SNP–treatment outcome associations. A post hoc power analysis was performed using G*Power version 3.1.9.7 (Heinrich Heine University Düsseldorf, Düsseldorf, Germany) based on the final sample size of 75 patients. Assuming a medium effect size (w = 0.30), an α level of 0.05, and one degree of freedom, the achieved statistical power was 73.83%. The study included all consecutive patients who fulfilled the eligibility criteria during the study period, resulting in a final sample of 75 patients.

## 5. Conclusions

Although the present study is limited by the relatively small number of participants, this should be interpreted in the context of progressive pulmonary fibrosis being a rare and clinically heterogeneous disease, where large, well-characterized cohorts are often difficult to obtain. Nevertheless, our findings highlight the potential relevance of genetic variability in modulating treatment response and adverse effects in patients receiving antifibrotic therapy. Further large-scale and multicenter studies are needed to validate these observations and to better define the role of pharmacogenetic markers in optimizing personalized therapeutic strategies for patients with PPF.

## Figures and Tables

**Table 1:** Baseline pulmonary function parameters according to disease progression at 1-year follow-up.

Variables	Patients with Progression at Year 1	Patients Without Progression at Year 1	*p*-Value
Age	67.5 (59.75–72)	64 (54–71.5)	0.25
FEV1 (%)	76.5 (68.25–84.25)	76 (62–93.25)	0.99
FEV1 (L)	1.74 (1.54–2.56)	1.96 (1.51–2.57)	0.55
FVC (%)	79.5 (66.75–89.5)	66 (59–90)	0.21
FVC (L)	2.13 (1.76–2.79)	2.26 (1.81–3.03)	0.68
FEV1/FVC (%)	82.93 (75.44–85.47)	82 (76.99–88.65)	0.54
DLCO (%)	38 (26.5–50.5)	38 (30.5–49.5)	0.65
KCO (%)	52 (45.5–67.75)	56 (44.5–65)	0.94
6MWT:			
HR before (/min)	89 (83.25–98)	83 (73.5–95.5)	0.16
HR during (/min)	111.5 (101.75–123)	104 (95.25–116.75)	0.09
HR after (/min)	90 (80.5–94.75)	85 (77–96.75)	0.53
SpO_2_ before (%)	96.5 (95.75–98)	97 (94–98)	0.68
SpO_2_ during (%)	88.5 (84–97.25)	89 (84.25–94.75)	0.6
SpO_2_ after (%)	97 (95.75–98)	96 (93.25–98)	0.54
Distance (m)	372 (288.75–405.75)	390 (304–440)	0.29
CT alveolar score	10.5 (5–12.25)	8 (6–10)	0.17
CT reticular score	18.5 (10.75–21.5)	14 (11–18)	0.15

FEV1—forced expiratory volume in one second; FVC—forced vital capacity; FEV1/FVC—ratio of forced expiratory volume in one second to forced vital capacity; DLCO—diffusing capacity of the lung for carbon monoxide; KCO—carbon monoxide transfer coefficient (DLCO corrected for alveolar volume); 6MWT—six-minute walk test; HR—heart rate; SpO_2_—oxygen saturation; CT—computed tomography; L—liters; %—percentage of the predicted value; m—meter.

**Table 2:** Pulmonary function parameters, follow-up data at 6 and 12 months stratified by disease progression status at 1 year.

Variables	Progressors	Non-Progressors	*p*-Value	Odds Ratio
FEV1 decline after 6 months (%)	9.19 (1.35–19.03)	0 (−5.88–4.1)	<0.001	
Decline of more than 10% of FEV1 in the first 6 months	38.1%	9.24%	0.006	6.03 (95% CI 1.69–21.56)
FEV1 decline after 12 months	16.51 (10.72–22.85)	−1.72 (−10.6–4.4)	<0.001	
Decline of more than 10% of FEV1 after the first 12 months	57.14%	14.81%	<0.001	7.67 (95% CI 2.44–24.09)
FVC decline after 6 months (%)	11.22 (6.05–21.02)	−2 (−11.49 −−1.57)	<0.001	
Decline of more than 10% of FVC in the first 6 months	57.14%	4.91%	<0.001	25.78 (95% CI 5.36–123.99)
FVC decline after 12 months (%)	16.53 (11.92–23.89)	−5 (−16.17–1.73)	<0.001	
DLCO decline after 6 months (%)	7.59 (−1.83–13.45)	4.59 (−4.89–13.47)	0.66	
Decline of more than 15% of DLCO in the first 6 months	16.67%	21.67%	1	0.72 (95% CI 0.14–3.72)
DLCO decline after 12 months (%)	6.25 (−1.19–38.84)	2.44 (−13.56–22.5)	0.22	
Decline of more than 15% of DLCO after the first 12 months	33.33%	36.67%	1	0.86 (95% CI 0.23–3.2)

FEV1—forced expiratory volume in one second; FVC—forced vital capacity; DLCO—diffusing capacity of the lung for carbon monoxide; %—percentage of the predicted value.

**Table 3:** Associations between the *PDGFRB*, *VEGFR2*, and *TGFB1* gene polymorphisms and disease progression after one year and AE in patients with PPF.

Polymorphisms	*n* (%)	Patients with AE	*p*-Values *	Patients with Progression at Year 1	*p*-Values *
*PDGFRB* rs2229562					
TT	35 (46.7)	17 (48.6)	0.244	6 (17.1)	0.247
CT	32 (42.7)	10 (31.3)		4 (12.5)	
CC	8 (10.7)	2 (25)		3 (37.5)	
T	102 (68)	44 (43.1)	0.11	16 (23.2)	0.49
C	48 (32)	14 (29.2)		10 (20.8)	
*PDGFRB* rs246395					
TT	42 (56)	18 (42.9)	0.563	7 (16.7)	0.37
CT	28 (37.3)	10 (35.7)		4 (14.3)	
CC	5 (6.7)	1 (20)		2 (40)	
T	112 (74.7)	46 (41.1)	0.339	18 (16.1)	0.62
C	38 (25.3)	12 (31.6)		8 (21.1)	
*PDGFRB* rs2302273					
GG	44 (58.7)	14 (31.8)	0.298	8 (18.2)	0.691
AG	28 (37.3)	14 (50)		4 (14.3)	
AA	3 (4)	1 (33.3)		1 (33.3)	
G	116 (77.3)	42 (36.2)	0.317	20 (17.2)	1.000
A	34 (22.7)	16 (47.1)		6 (17.6)	
*VEGFR2* rs1870377					
TT	44 (58.7)	13 (29.5)	0.154	8 (18.2)	0.496
AT	25 (33.3)	13 (52)		5 (20)	
AA	6 (8)	3 (50)		0 (0)	
T	113	39 (34.5)	0.081	21 (18.6)	0.619
A	37	19 (51.4)		5 (13.5)	
*VEGFR2* rs2071559					
AA	19 (25.3)	7 (36.8)	0.846	3 (15.8)	0.642
AG	38 (50.7)	14 (36.8)		8 (31.1)	
GG	18 (24)	8 (44.4)		2 (11.1)	
A	76 (50.7)	28 (36.8)	0.738	14 (18.4)	0.830
G	74 (49.3)	30 (40.5)		12 (16.2)	
*TGFB1* rs1800470					
AA	21 (28)	8 (38.1)	0.930	2 (9.5)	0.389
AG	45 (60)	17 (37.8)		10 (22.2)	
GG	9 (12)	4 (44.4)		1 (11.1)	
A	87 (58)	33 (37.9)	0.866	14 (16.1)	0.667
G	63 (42)	25 (39.6)		12 (19)	

* At the level of significance of 0.05, according to Chi-square test or Fisher’s exact test. AE—adverse effects.

## Data Availability

Dataset available on request from the authors.

## Supplementary Materials

The following supporting information can be downloaded at: https://www.mdpi.com/article/10.3390/ijms27177546/s1.

## Abbreviations

The following abbreviations are used in this manuscript:

6MWT	six-minute walk test
AE	adverse events
ALT	alanine aminotransferase
AST	aspartate aminotransferase
ATP	adenosine triphosphate
ATS	American Thoracic Society
CI	confidence interval
CT	computed tomography
CTGF	connective tissue growth factor
CYC	cyclophosphamide
DLCO	diffusing capacity of the lung for carbon monoxide
DNA	deoxyribonucleic acid
DSP	desmoplakin
EGFR	epidermal growth factor receptor
ERS	European Respiratory Society
FEV1	forced expiratory volume in one second
FGF	fibroblast growth factor
FGFR	fibroblast growth factor receptor
FVC	forced vital capacity
GI	gastrointestinal
HCC	hepatocellular carcinoma
HLA	human leukocyte antigen
HRCT	high-resolution computed tomography
HWE	Hardy–Weinberg equilibrium
IgG	immunoglobulin G
IL	interleukin
ILD	interstitial lung disease
IPF	idiopathic pulmonary fibrosis
JRS	Japanese Respiratory Society
KCO	transfer coefficient of the lung for carbon monoxide
KDR	kinase insert domain receptor
mRNA	messenger RNA
MMF	mycophenolate mofetil
MUC5B	mucin 5B
OR	odds ratio
PCR	polymerase chain reaction
PDGF	platelet-derived growth factor
PDGF-BB	platelet-derived growth factor-BB
PDGFR	platelet-derived growth factor receptor
PDGFRB	platelet-derived growth factor receptor beta gene
PPF	progressive pulmonary fibrosis
SARD-ILD	systemic autoimmune rheumatic disease-associated interstitial lung disease
SNP	single-nucleotide polymorphism
SSc	systemic sclerosis
SSc-ILD	systemic sclerosis-associated interstitial lung disease
TGFβ	transforming growth factor beta
TGFB1	transforming growth factor beta 1 gene
UTR	untranslated region
VEGF	vascular endothelial growth factor
VEGFR	vascular endothelial growth factor receptor
VEGFR2	vascular endothelial growth factor receptor 2
