# Supplementary material for: Association of PDGFRB, VEGFR2, and TGFB1 Gene Polymorphisms with Nintedanib Efficacy and Adverse Effects in Patients with Progressive Pulmonary Fibrosis
Source: Int J Mol Sci. 2026 Aug 23;27(17):7546. doi: 10.3390/ijms27177546 (PMC13565948; doi:10.3390/ijms27177546)
Supplement: Supplementary file 1 [file ijms-27-07546-s001.zip › ijms-4494813-supplementary.pdf]

Supplementary Table S1. Genomic characteristics, allele frequencies, and functional relevance of the analyzed *PDGFRB*, *VEGFR2*, and *TGFB1* polymorphisms.

Gene	dbSNP ID	HGVS nomenclature (GRCh38.p14)	Chromosomal position (GRCh38.p14)	Gene region	Reference allele	Alternative allele	European MAF	Functional relevance / predicted effect	Reference
PDGFRB	rs2302273	NC_000005.10:g.150155692G>A	chr5:150155692	5′UTR	G	A	0.21 (A)	Cis-acting factor.	Volz, et al. 2015. [41]
PDGFRB	rs246395	NC_000005.10:g.150120109T>C	chr5:150120109	Exon 19	T	C	0.33 (C)	Synonymous variant. Effect on protein level.	Estevez-Garcia et al. 2012. [57].
PDGFRB	rs2229562	NC_000005.10:g.150115690T>C	chr5:150115690	3′UTR	T	C	0.43 (C)	/	/
VEGFR2/ KDR	rs1870377	NC_000004.12:g.55106807T>A	chr4:55106807	Exon 11	T	A	0.24 (A)	p.Gln472His. Effect on VEGF binding to VEGFR2.	Wang et al. 2007. [61]
VEGFR2/ KDR	rs2071559	NC_000004.12:g.55126199A>G	chr4:55126199	2KB Upstream variant	A	G	0.50 (G)	E2F binding site effect on transcription and expression.	Wang et al. 2007. [61]
TGFB1	rs1800470	NC_000019.10:g.41353016G>A	chr19:41353016	Exon 1	G	A	0.49 (G)	p.Leu10Pro. Effect on secretion and function.	Gu et al. 2012. [62]

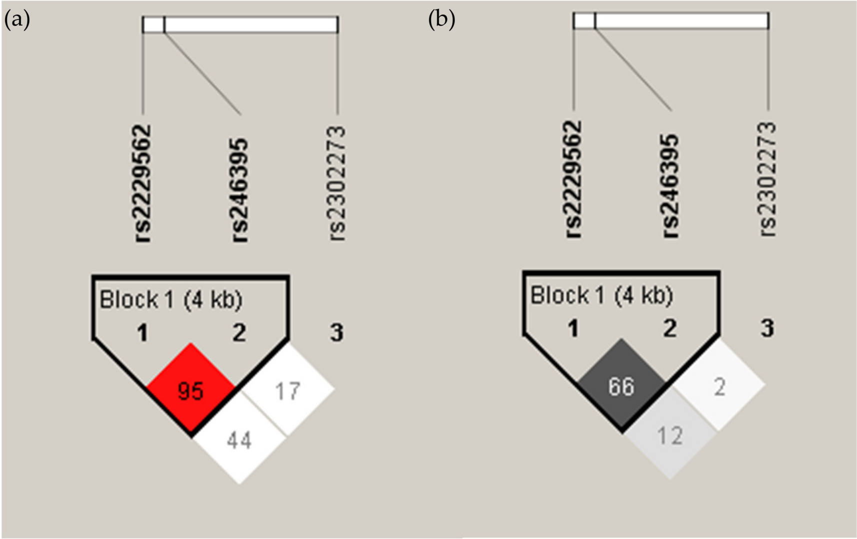

Supplementary Figure S1. Schematic representation of linkage disequilibrium (LD) with D′ (a) and r^2 (b) values among the analyzed *PDGFRB* gene polymorphisms (rs2229562, rs246395, and rs2302273) in patients with progressive pulmonary fibrosis (PPF).

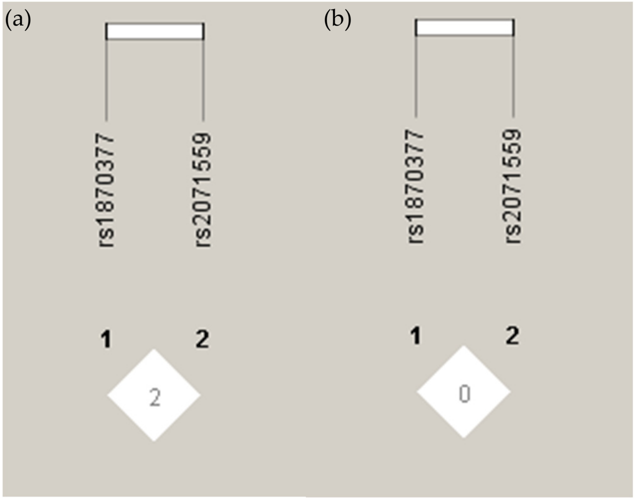

Supplementary Figure S2. Schematic representation of linkage disequilibrium (LD) with D′ (a) and r^2 (b) values among the analyzed *VEGFR2* gene polymorphisms (rs1870377 and rs2071559) in patients with progressive pulmonary fibrosis (PPF).

Supplementary Table S2. Frequencies of *PDGFRB* haplotypes (rs2229562, rs246395, and rs2302273) according to treatment efficacy and the occurrence of adverse events in patients with progressive pulmonary fibrosis.

Haplotype	Frequency (%)	1-year FVC decline <10%	1-year FVC decline ≥10%	p - value	Without AE	With AE	p - value	Without GI AE	With GI AE	p - value	Without diarrhea	With diarrhea	p - value
TTG	59.7	58.2	64.3	0.748	56.7	64.7	0.416	59.7	60.1	0.869	59.9	59.9	0.901
CCG	15.4	16.9	10.7	0.648	20.1	7.7	0.059	18.8	7.3	0.115	18.5	5.9	0.083
CCA	9.3	6.8	17.9	0.174	8.1	11.3	0.664	7.1	14.4	0.256	8.3	12.5	0.537
TTA	7.6	9.8	<1%	0.204	6.3	9.4	0.322	6.6	9.5	0.323	6.2	11.2	0.218
CTA	5.1	5.5	3.6	0.68	5.1	5.2	0.954	4.5	6.5	0.665	4.2	7.9	0.419
CTG	2.2	1.9	3.6	0.587	3.6	<1%	0.182	3.2	<1%	0.276	2.9	<1%	0.35
TCA	<1%	<1%	<1%	<1%	<1%	1.7	NA	<1%	2.2	NA	<1%	2.6	NA

FVC – forced vital capacity; AE – adverse events; GI AE – gastrointestinal adverse events; NA – not estimable because the haplotype was absent or occurred at an insufficient frequency in the respective subgroup to perform the comparison.

Supplementary Table S3. Frequencies of *VEGFR2* haplotypes (rs1870377 and rs2071559) according to treatment efficacy and the occurrence of adverse events in patients with progressive pulmonary fibrosis.

Haplotype	Frequency (%)	1-year FVC decline <10%	1-year FVC decline ≥10%	p - value	Without AE	With AE	p - value	Without GI AE	With GI AE	p - value	Without diarrhea	With diarrhea	p - value
TA	38.5	37.5	35.7	0.578	41.8	33.3	0.272	42.6	26.5	0.137	42.4	25.3	0.119
TG	36.9	36.2	46.4	0.79	38.6	34	0.6	36.2	40.9	0.875	36.2	40.5	0.873
AG	12.5	13.8	<1%	0.365	9.2	17.7	0.142	10.9	13.5	0.315	11.1	14.8	0.295
AA	12.2	12.5	17.9	0.759	10.4	15	0.357	10.3	19.2	0.336	10.3	19.5	0.306

LFVC – forced vital capacity; AE – adverse events; GI AE – gastrointestinal adverse events; NA – not estimable because the haplotype was absent or occurred at an insufficient frequency in the respective subgroup to perform the comparison.
